# The Impact of Blockchain Application on the Qualification Rate and Circulation Efficiency of Agricultural Products: A Simulation Analysis with Agent-Based Modelling

**DOI:** 10.3390/ijerph19137686

**Published:** 2022-06-23

**Authors:** Liukun Wang, Chunjie Qi, Peng Jiang, Si Xiang

**Affiliations:** College of Economics & Management, Huazhong Agricultural University, Wuhan 430070, China; liukunwang@webmail.hzau.edu.cn (L.W.); peng.jiang1@outlook.com (P.J.); xiangsi0123@126.com (S.X.)

**Keywords:** blockchain, agricultural products, qualification rate, circulation efficiency, simulation analysis, food health

## Abstract

In the context of COVID-19, the circulation of agricultural products is increasingly important for the nutrition and health of people. With the changing needs of society and the advancement of technology, the agricultural product circulation system needs to undergo corresponding changes to adapt to the modern fast-paced social system. Blockchain technology couples with the circulation of agricultural products, as its technical features, such as immutability and a distributed ledger database, ensures the speed and stability of the key information circulation process of agricultural products. The research goal of this paper was to clarify the influence of blockchain technology on the qualification rate and circulation efficiency for agricultural products. Based on the main characteristics of blockchain technology and a summary of domestic and foreign theoretical research, this paper simulated the impacts of blockchain technology on the agricultural product circulation system. The results revealed that blockchain technology can improve the qualification rate of agricultural products and thereby ensure their quality and safety. The introduction of blockchain increased the qualification rate by nearly 30%. Moreover, blockchain technology significantly enhanced the efficiency of the agricultural product circulation system, thereby greatly promoting economic benefits. The introduction of blockchain increased circulation efficiency by nearly 15%. Finally, the introduction of blockchain technology can effectively promote the governance level and reduce the supervision costs of the agricultural product circulation system. Through simulation analysis, we found that blockchain technology has a positive impact on both the qualification rate and circulation efficiency for agricultural products. These findings enrich research into the application of blockchain technology in the management and circulation of modern agricultural products.

## 1. Introduction

Circulation is the intermediary bridge between commodity production and consumption [1,2]. In China, great attention has been paid to the production of commodities, while the circulation of commodities has been largely ignored. In recent years, there have been sharp fluctuations in the price of vegetables, such as spinach, and the problem that has been described as “cheap vegetables hurt farmers and expensive vegetables hurt the customers” has become increasingly prominent [3,4]. Moreover, the frequent occurrence of quality and safety problems with agricultural products, such as “melamine” in Sanlu milk powder and “clenbuterol” in pork, has posed great threats to people’s health and safety, as well as a serious crisis of trust [5,6,7]. In the context of COVID-19, the circulation of agricultural products is increasingly important for the nutrition and health of people. Therefore, the low quality, safety, and circulation efficiency for agricultural products are urgent problems in China [8,9]. The core consideration in solving these three problems is improvement in the qualified rate and circulation efficiency for agricultural products.

The newly emerging digital technologies represented by blockchain, 5G, artificial intelligence, and cloud computing, which are prevalent in all aspects of the economy and society, are symbols of the world economy’s next Kondratieff cycle [10,11,12]. However, research on the application of blockchain technology to agriculture is still in its initial stage [13,14]. Blockchain technology has promising prospects for application in the circulation industry [15], as its technical features, such as immutability and a distributed ledger database, can ensure the speed and stability of the key information circulation process for agricultural products, which may solve the urgent problems of agricultural product circulation in China. However, current research on blockchain technology is mostly focused on the circulation of industrial products, while little research has been concerned with its application in the circulation of agricultural products.

Recently, a nascent strand of literature has studied the effects of blockchain technology. For example, some studies discussed the impact of the informatization level on the productivity of the circulation industry in 30 provinces of China, and found that blockchain technology can improve the circulation efficiency in both a local area and its adjacent areas [16,17].

Existing studies mainly focused on the following three aspects of blockchain technology. (1) A traceability system for agricultural products, e.g., a consortium blockchain-based model for agricultural traceability systems, was designed to address a system’s vulnerability to cyberattacks due to the presence of a hub [18]. Some scholars analyzed the root causes of agricultural product safety problems and theoretically investigated the role of the network system of blockchain technology in solving the safety problems of agricultural products [5]. (2) Some existing studies provided a theoretical framework and case studies on rural finance, in support of a proposal that blockchain technology breaks the information barriers of traditional agricultural supply chains and reduces financial institutions’ difficulty in accurately verifying information [19]. Other scholars revealed that blockchain and agricultural crowdfunding can contribute to significant integration and innovation, which can reduce the difficulty of agricultural crowdfunding at the theoretical level, optimize the operational process, and reduce the risk of agricultural crowdfunding at the practical level [20]. (3) Some studies explored agricultural production and operation methods to assess the role of blockchain technology in the circulation industry from the perspective of the information technology and supply chain, and demonstrated that the application of blockchain technology can facilitate the development of the circulation industry [21,22,23,24]. Other studies explored the construction of port circulation from the perspective of informatization and suggested that the rapid development of high-tech represented by informatization has shaped a new type of cross-border e-commerce, which has a large impact on the port circulation industry [25,26,27].

We found relatively few studies on the application of blockchain technology to the circulation of agricultural products; however, there is agreement in existing studies on how information technology can improve the efficiency of agricultural circulation [28,29]. Blockchain technology has the technical characteristics of information sharing, real and secure data, information and certificate traceability, smart contract, etc. [30,31]. There are multiple couplings between blockchain technology and the demands of agricultural products’ circulation [32]. 

The core consideration in determining the role of blockchain technology in the circulation system of agricultural products lies in the breakthrough of circulation efficiency, supported by a new generation of information technology and adapted to the changes in supply and demand in the new digital era. These changes provide a potential mechanism for insight into consumer needs, promoting the matching of supply and demand and linking the reallocations of production [33,34].

However, research on the application of blockchain technology to the agricultural circulation system is still in its initial stage [13]. The most urgent problem for blockchain technology in improving the circulation system of agricultural products is to determine “how will blockchain technology affect the pass rate and circulation efficiency of agricultural products?”. Little research has been done to integrate blockchain technology with the quality and safety of agricultural products and the efficiency of agricultural circulation. The qualification rate reflects the quality and safety level of agricultural products. The circulation efficiency for agricultural products refers to the overall efficiency of each link in the whole process, from production to consumption. Most of the existing studies evaluated the circulation efficiency for agricultural products from the perspective of input and output [35,36]. This paper adopts a simulation that combines the input-output ratio and the product qualification rate to determine the circulation efficiency for agricultural products, and carries out a standardized process. This paper’s research goal is to clarify the influence of blockchain technology on the qualification rate and the circulation efficiency for agricultural products.

To address the research gaps, this paper combines modern circulation theory and the characteristics of blockchain technology to simulate the impacts of blockchain technology on the qualification rate and the circulation efficiency for agricultural products, extends the research on the blockchain technology in the circulation system of agricultural products, and provides a reference for other scholars in helping to promote the modernization, transformation, and upgrading of the agricultural product circulation system.

This paper contributes to the literature in three ways. First, this study extends the theoretical framework of blockchain theory to the study of the circulation of agricultural products. Previous studies mainly focused on the impact of the circulation subject, the circulation object, and the circulation carrier on the efficiency of agricultural products’ circulation [37,38]; however, few studies devoted attention to the impact of blockchain technology on agricultural products’ circulation system. Second, this study constructs a simulation model to measure the influence of blockchain technology on the qualification rate and the circulation efficiency for agricultural products. Finally, this study considers the dynamic changes in the circulation efficiency and the qualified rate for agricultural products under different supervision intensities.

The remainder of this paper is organized as follows. In Section 2, we provide a theoretical analysis framework for the effect of block chain technology on the circulation system of agricultural products and suggest hypotheses for application. In Section 3, we construct the simulation model with agent-based modelling. In Section 4, we provide the results of the simulation analysis of the qualification rate of agricultural products with a descriptive statistical analysis; the results of the simulation of the circulation efficiency for agricultural products; and the impact of regulatory intensity on the circulation efficiency and qualification rate for agricultural products. Finally, Section 5 provides the conclusions and policy implications of this study.

## 2. Theoretical Analysis and Research Hypotheses

As an emerging digital technology, blockchain has been developing rapidly since 2008, and has been applied gradually in various fields of the economy and society due to its advantages of immediacy, decentralization, immutability, and traceability [39,40]. 

Based on previous studies, the main agents of the agricultural product circulation system in China can be divided into farmers, wholesalers, retailers, consumers, and regulators [41]. Due to the technical characteristics of blockchain, it can only work in the agricultural circulation system if all of these main agents use blockchain technology for uploading information [42]. With the use of blockchain technology, all of these agents can consider the information in the blockchain database [43].

**Figure 1:**
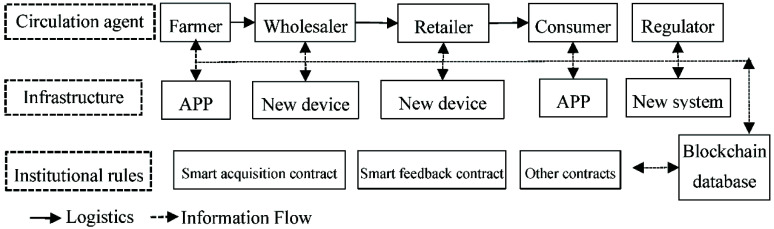
Operation diagram of blockchain technology in the agricultural product circulation system.

When blockchain technology is applied in the agricultural product circulation system, the blockchain-based IoT device deployed by various circulation agents can collect the key data, such as information on production, transport, processing, warehousing, and sales, and upload the relevant information in the blockchain database via algorithms for screening and calculation. Blockchain technology has a positive impact on the agricultural product circulation system by improving the infrastructure and the institutional rules for different agents in the system (Figure 1).

For farmers, the IoT devices based on blockchain technology can collect information on their production processes, which can be uploaded and queried in the blockchain database. Farmers can release their products on the relevant APP or Internet platform and track orders in real time. When the sales process is completed, the agricultural products are transferred from farmers to consumers, and the smart contracts based on blockchain technology automatically complete the order.

Wholesalers are mainly involved in the transport, processing, and warehousing of agricultural products. In the transport process, intelligent equipment equipped with transport vehicles collect information, such as the transport time and the number and plots of agricultural products, and upload the information onto the database. In processing, the sensor equipment that is configured in the processed products and the IoT equipment that is carried by the workers can collect the key data for agricultural product processing in real time and upload it to the database. In the warehousing process, intelligent equipment installed in the warehouse collects data, such as the storage time, the plot, and the producers of products, and uploads the data. After mutual verification of the data in each process, a time stamp, a location stamp, and a quality stamp are generated for each unit of the agricultural products. The series of actions are completed by wholesalers equipped with blockchain equipment.

Through blockchain technology, consumers can purchase traceable agricultural products with unique time stamps, location stamps, and quality stamps and obtain important information for each step in the circulation process of the agricultural products, effectively solving the problem of information asymmetry between buyers and sellers. At the same time, consumers pay more for such products because of the increased cost of the technology input.

Because the whole process of agricultural product circulation adopts blockchain and IoT technology and the information on each process and agent are stored in the database, regulators can conduct real-time supervision of every agent and every step in the circulation system. In this way, unsafe products can be traced back to the production stage, and relevant agents will be accountable.

Based on the above theoretical analysis, two hypotheses for blockchain technology’s effects on the circulation system of agricultural products are proposed:

**Hypothesis 1** **(H1).** *When blockchain is applied, the qualification rate of agricultural products will be further improved*.

**Hypothesis 2** **(H2).** *When blockchain is applied, the circulation efficiency of agricultural products will be significantly improved.*

## 3. Methodology

### 3.1. Simulation with Agent-Based Modelling

As the application of blockchain technology in agriculture is in its infancy, the available macro data is very limited. Therefore, in this article, we study the influence of block chain technology on the qualification rate and circulation efficiency for agricultural products via a simulated analysis.

A simulation analysis with agent-based modelling is a bottom-up modeling method. It describes the behaviors of individual agents with each other, observes the emergence of the overall actions of the agents, and determines macro laws to explain specific phenomena in the real world and describe the macro behaviors of complex systems [44]. In this method, the modeling object is defined according to the different individual components in the system, the key attributes are abstracted to establish the individual agent model, and the agent attributes, behaviors, interaction rules, and related constraints are established. Through the operation of this system, the influence of blockchain technology on the qualification rate and circulation efficiency of the agricultural circulation system is studied.

### 3.2. Agents

Based on theoretical analysis, we established five agents in the simulation model: farmers, wholesalers, retailers, consumers, and regulators. According to the assumption made in economics of “economic man”, all agents pursue their economic interests. The interests of consumers in the model are focused on health, so consumers prefer healthier agricultural products and are willing to pay higher prices for it such products The economic behavior and transformation strategy of each agent, as well as the behavior rules that operate between agents, are discussed in the next section.

### 3.3. Simulation Analysis

Netlogo version 6.2 (Uri Wilensky, The United States) provides complete simulation rules through programming. Netlogo is not constrained by established rules and provides a high degree of freedom. The data can be exported to a CVS format that can be read by Excel, and the data can be diagrammed directly so that users can view the data changes directly during simulation [45]. Therefore, Netlogo version 6.2 is suitable for simulating the specific scenario of introducing the agricultural products’ circulation system by blockchain technology.

First, based on the field research and theoretical analysis, we established the behavior rules for each subject. Second, we developed codes to simulate the circulation system of agricultural products in Netlogo version 6.2. Third, we ran the model in software and observed how it worked. Finally, we constantly adjusted and modified the model to make the simulation experiment more credible.

## 4. Construction of the Simulation Model

In recent years, agent-based simulation has been widely used in the field of social science, where it is effective in research on the formation and evolution of organizations [46,47]. Therefore, on this basis, we constructed an agent-based simulation model, in which blockchain technology is applied to the agricultural product circulation system. The blockchain technology involved in this article is a consortium chain, contrary to public and private chains. The simulation model is constructed as set out below.

### 4.1. Model Structure

**Figure 2:**
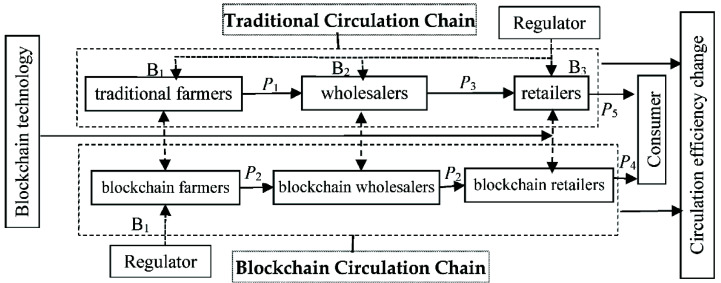
Frame diagram of the simulation model of the agricultural product circulation system.

The simulation model includes farmers, wholesalers, retailers, consumers, and regulators, as well as the interactive behaviors among these five agents, and considers both the traditional circulation chain and the blockchain circulation chain (Figure 2).

Assuming that there is an agricultural product production base in the initial state, the production base consists of “n” homogeneous farmers, each of whom independently produces “s” units of agricultural products in each period. The products produced by farmers are classified into two types: safe and unsafe (unsafe products refer to products whose pesticide residues do not meet national standards). Because quality and safety are important attributes of credible products, only the farmers know the true quality of the products and other agents cannot identify them. At the same time, there are “m” wholesalers and “k” retailers. Under the initial conditions, agricultural products are only circulated through the traditional circulation chain. In this mode, the products are sequentially circulated from traditional farmers to wholesalers, retailers, and consumers, from whom the farmers, wholesalers, and retailers make profits based on the different stages. After the application of blockchain technology by some farmers, wholesalers, and retailers, two chains in the agricultural product circulation system result. In the blockchain circulation chain, the blockchain farmers conduct transactions directly with the blockchain retailers, and the blockchain wholesalers mainly undertake the function of logistics. The farmers, wholesalers, and retailers can switch between the traditional mode and the blockchain mode, under certain conditions.

### 4.2. Setting of the Farmer Agent

In the traditional circulation chain, agricultural products produced by farmers are first sold to wholesalers, and then sold to retailers. After the application of blockchain technology, the blockchain farmers sell the products directly to the blockchain retailers. The blockchain wholesalers mainly undertake the transport function. According to the assumption in economics of “economic man”, farmers are profit-seeking, with attributes of identity, honesty, and wealth [35]. We assume that farmers always seek higher economic benefits and that profits have the greatest influence on their actions and decisions. The specific decision-making behavior and conversion rules of farmers are set out below.

Honesty updating:

Each farmer has a credibility rating, and the change in its value can indicate whether the farmer produces safe products as well as the farmer’s resistance to the impact of competing interests. The benefit impact refers to the difference in the expected benefits from the production of safe and unsafe products. If traditional farmers and blockchain farmers are not sampled for inspection, their honesty factor will be slightly reduced according to Equation (1). If traditional or blockchain farmers are sampled for inspection by the regulator, their honesty factor will be increased based on Equation (2). If any of the blockchain farmers, blockchain wholesalers, and blockchain retailers are sampled by the regulator, the honesty factor of the blockchain farmers will be increased according to Equation (2). If the traditional and blockchain farmers, blockchain wholesalers, and blockchain retailers are not sampled by the regulator, the honesty factor of the farmers is decreased according to Equation (1).


(1) $$Honesty_{t+1}=\left(1-f^{+}\right)Honesty_{t}+minimal\_Honesty$$

(2) $$Honesty_{t+1}=\left(1-f^{-}\right)Honesty_{t}+f^{-}$$

In these equations, honesty represents the honesty of a certain farmer in period *t*. The minimum value of honesty is 0, when the farmer is completely dishonest and all his or her products are unsafe. When honesty is 1, the farmer is completely honest, and correspondingly all his or her products are safe. As honesty increases, the probability of the farmer producing safe products increases. $f^{+}\in \left(0,1\right)$ and $f^{-}\in \left(0,1\right)$ represent the positive and negative impact factors experienced by the farmer, respectively, and if $f^{-}>f^{+}$, the negative factor has the greater impact on the farmer [46]. Minimal_Honesty is the minimum value of honesty.

Punishment:

There are situations in which farmers will be punished. When the regulators detect some problems in agricultural products during the blockchain circulation, they can directly trace the source through blockchain technology [34] to identify the blockchain farmers who produce unsafe products and impose certain penalties on them. In addition, when the regulators find some problems in the agricultural products of traditional farmers by spot checks, the traditional farmers will be punished.

Farmers’ production decisions:

Farmers in the production base have two choices: producing safe products or producing unsafe products. Profit, honesty, and other random factors jointly determine the production decisions of farmers, as shown in Equation (3).

(3) $$\alpha k_{i}+\left(1-\alpha \right)p>Honesty$$

In Equation (3), $k_{i}\in \left(0,1\right)$ represents the standardized value of the difference between the two expected benefits of producing safe and unsafe products. In the weights α∈(0,1), ρ is any random number on (0,1). If this equation is satisfied, the farmers will produce safe products; otherwise, they will produce unsafe products.

Product-trading decisions: 

Traditional farmers sell their products to wholesalers at a price of *P*_1_, and the profit of the farmer is the income minus the production costs (with any penalty deducted). Blockchain farmers sell their products to the blockchain retailers at price *P*_2_, and the profit is the income minus the production and technology costs. Profits will change the attributes of farmers’ wealth. If a farmer sells all of his or her products in the applicable period, regardless of whether the products are safe or unsafe, the transactions between the farmer and the wholesalers are conducted at a consistent price *P*_1_. Because of blockchain technology, the blockchain farmers must be responsible for the quality and safety of the agricultural products in the whole process; as a result, *P*_2_ is generally higher than *P*_1_. *P*_3_ is the price at which the wholesalers sell agricultural products to retailers. *P*_4_ and *P*_5_ are the prices of agricultural products sold to consumers by blockchain retailers and traditional retailers, respectively. The calculation of *P*_1_, *P*_2_, *P*_3_, *P*_4_, and *P*_5_ can be determined with Equations (4)–(8). In these equations, $\varepsilon \left(t\right)\sim N\left(\mu ,\;\;\sigma^{2}\right)$ is the random item and e is the effect of market conditions on prices.$\;C_{s}$ and $C_{u}$ indicate the unit cost of producing a safe product and an unsafe product, respectively; η∈(0,1), β∈(0,1), δ∈(0,1).

(4) $$P_{1}\left(t\right)=\alpha C_{u}+\left(1-\alpha \right)C_{s}+\varepsilon_{1}\left(t\right)+e_{1}$$

(5) $$P_{2}\left(t\right)=\alpha C_{u}+\left(1-\alpha \right)C_{s}+\varepsilon_{2}\left(t\right)+e_{2}$$

(6) $$P_{3}\left(t\right)=\left(1+\eta \right)P_{1}\left(t\right)+e_{3}$$

(7) $$P_{4}\left(t\right)=\left(1+\beta \right)P_{2}\left(t\right)+e_{4}$$

(8) $$P_{5}\left(t\right)=\left(1+\delta \right)P_{3}\left(t\right)+e_{5}$$

If the agricultural products produced by the farmers are determined to be unsafe by the regulator, they will be fined *B1* per unit, and their honesty will be changed accordingly. For traditional farmers, there are three profit functions:

$\varphi_{1}=s\left(P_{1}-C_{u}\right)$: farmers produce unsafe products that are not sampled.

$\varphi_{2}=s\left(P_{1}-C_{u}-B_{1}\right)$: farmers produce unsafe products that are detected by regulators.

$\varphi_{3}=s\left(P_{1}-C_{s}\right)$: farmers produce safe products.

*C_t_* is the cost of introducing blockchain technology. For blockchain farmers, there are also three profit functions:

$\pi_{1}=s\left(P_{2}-C_{u}-C_{t}\right)$: blockchain farmers produce unsafe products that are not detected.

$\pi_{2}=s\left(P_{2}-C_{u}-C_{t}-B_{1}\right)$: blockchain farmers produce unsafe products that are detected by regulators.

$\pi_{3}=s\left(P_{2}-C_{s}-C_{t}\right)$: blockchain farmers produce safe products.

Conversion strategy: 

Farmers have certain adaptability in their pursuit of maximum profits, different types of farmers can be converted to each other. Accordingly, the following issues need to be considered in the conversion. The conditions for conversion are mainly based on a comparison of the average incomes of different types of farmers; the threshold for conversion (*G*) reflects the resistance of the conversion [48]. When Equation (9) is satisfied, the type of the farmer will be converted; otherwise, the farmer type will not be changed.

(9) $$\alpha \left(\pi_{i}^{\prime }-\pi_{i}\right)/\pi_{i}^{\prime }+\left(1-\alpha \right)p>G$$

In Equation (9), $\pi_{i}$ is the current profit of the farmer; $\pi_{i}^{\prime }$ is the current average profit of different types of farmers around the farmer; p∈(0,1); *G* is the conversion threshold, and its dynamic change can be expressed as follows:

(10) $$G_{t+1}=\left(1-v_{1}^{+}\right)G_{t}+v_{1}^{+}$$

(11) $$G_{t+1}=\left(1-v_{1}^{-}\right)G_{t}\;\;$$

(12) $$G_{t+1}=\left(1-v_{2}^{+}\right)G_{t}+v_{2}^{+}$$

(13) $$G_{t+1}=\left(1-v_{2}^{-}\right)G_{t}$$

$v_{2}^{-}>v_{1}^{-}>v_{1}^{+}>v_{2}^{+}$. $v_{1}^{+}$ indicates that the personal income of the farmer is greater than the average income of different types of farmers, exclusive of fines. In this case, the farmer’s conversion threshold will be significantly increased. $v_{1}^{-}$ means that the farmer’s personal income is low, exclusive of fines; with fines, the conversion threshold will be slightly decreased. $v_{2}^{+}$ means that the personal benefit is high, but a fine is imposed, in which case the conversion threshold will be slightly increased; $v_{2}^{-}$ means that the farmer has a low income with a fine, when the conversion threshold will be significantly decreased.

### 4.3. Setting of the Wholesaler Agent

We assume that wholesalers make the following major decisions and carry out the following behaviors, each of which is intended to obtain greater economic benefits.

Product acquisition and sales decisions: 

The blockchain wholesaler obtains a fixed income θ per unit by providing logistical services for the blockchain circulation. The price of agricultural products purchased by traditional wholesalers is *P*_1_, and the price of of agricultural products sold to traditional retailers is *P*_3_.

For traditional wholesalers, there are two scenarios for their profit margins, *r*_1_ and *r*_2_.

$r_{1}=P_{3}-P_{1}$*,* when the agricultural product has not been tested or has passed the test.

$r_{2}=P_{3}-P_{1}-B_{2}$, when the agricultural products are detected as unsafe.

Punishment:

When the agricultural products in the circulation of the wholesaler are detected as unsafe, there are two situations. First, the products transported by the blockchain wholesalers are detected as unsafe, and the unsafe products can be traced through blockchain technology. In this case, the blockchain farmers producing the products will be punished. Second, the products of traditional wholesalers are detected as unsafe. In this case, the regulator will impose a penalty of B_2_ per unit on traditional wholesalers.

Conversion strategy:

Wholesalers also obey the assumption of the “economic man”. After the introduction of blockchain technology, different types of wholesalers can be converted to each other, but the following conditions need to be considered. The premise of conversion is that there are different types of wholesalers in the circulation system. The conversion conditions are mainly based on a comparison of the average incomes of different wholesaler types. Conversion threshold *G*_1_ is used to evaluate the resistance to conversion. When Equation (14) is satisfied, the type of wholesalers will be converted.

(14) $$\alpha \left(r_{i}^{\prime }-r_{i}\right)/r_{i}^{\prime }+\left(1-r\right)p>G_{1}$$

In Equation (14), $r_{i}$ is the current profit margin of a wholesaler; $r_{i}^{\prime }$ is the average profit margin of another type of wholesaler; α∈(0,1), p∈(0,1) and *G*_1_ is the conversion threshold.

### 4.4. Setting of the Retailer Agent

We assume that retailers make the following major decisions and carry out the following behaviors, each of which is intended to obtain greater economic benefits.

Product acquisition and sales decisions: 

Under the initial conditions, all retailers buy agricultural products from wholesalers. After the introduction of blockchain technology, some traditional retailers are converted into blockchain retailers. There are two situations at this time. First, the blockchain retailers directly purchase agricultural products at the price of *P*_2_ from the blockchain farmers and pay the transport cost per unit θ of the blockchain circulation, and then sell the products to consumers at the price of *P*_4_. Second, traditional retailers buy agricultural products from traditional wholesalers at the price of *P*_3_ and sell them to consumers at the price of *P*_5_.

At this time, the profit margin function of the blockchain retailer is $y_{1}=P_{4}-P_{2}-\vartheta -C_{t}$, and the profit margin function of a traditional retailer is $y_{2}=P_{5}-P_{3}$ when the product is not found to be unsafe by random inspection and $y_{3}=P_{5}-P_{3}-B_{3}$ when the product is sampled and found to be unsafe.

Punishment: 

When the blockchain retailer’s products are detected as unsafe, the source of the products can be traced through blockchain technology and the blockchain farmers who produce unsafe agricultural products will be fined B_1_ per unit. When the traditional retailer’s products are detected as unsafe, the regulator will impose a fine of B3 per unit.

Conversion strategy: 

The retailers also have the characteristics of the “economic man”, and thus different types of retailers can be converted to each other. The following factors need to be considered for the conversion. The premise for the conversion is that after the introduction of blockchain technology, there are different types of retailers. The conversion conditions are based on a comparison of the average incomes of different types of retailers. The conversion threshold *G*_2_ reflects the resistance to conversion. When Equation (15) is satisfied, the type of retailers will be converted.

(15) $$\alpha \left(C_{i}^{\prime }-C_{i}\right)/C_{i}^{\prime }+\left(1-\alpha \right)p<G_{2}$$

In Equation (15), $C_{i}$ is the current profit margin of a retailer; $C_{i}^{\prime }$ is the average profit margin of another type of retailer; α∈(0,1), p∈(0,1) and *G*_2_ is the conversion threshold.

### 4.5. Setting of the Regulator Agent

In the initial state, we assume that the sampling frequency of the supervisor for the farmers, wholesalers, and retailers is *a1*, *a2*, and *a3,* respectively, and the fine for unsafe products is *B1*, *B2*, and *B3* per unit, respectively. After the introduction of blockchain technology, the supervision of the traditional circulation chain remains unchanged. In the blockchain circulation chain, any unsafe products randomly detected in any link can be traced back to the blockchain farmers, and correspondingly a fine of B1 per unit is imposed on the farmer.

## 5. Simulation Experiments and Results

Based on the above simulation model, we used the Netlogo version 6.2 to simulate the agricultural product circulation system, and simulation experiments were carried out to explore the dynamic changes in the system under the application of blockchain technology. The parameters for the simulation experiments were derived from real cases, field investigations, and related studies [49]. In order to ensure the reliability of the simulation results, we added market environment and other parameters to the simulation process and set the transformation threshold to a range quantity.

After the introduction of blockchain technology, we observed dynamic changes in the product qualification rate and the circulation efficiency in the blockchain circulation chain and traditional circulation chain. The circulation efficiency was calculated from the product qualification rate and the input-output ratio of the circulation process according to a certain weighting. The data obtained from the simulation experiments were all generated by the simulation system.

### 5.1. Simulation Analysis of Qualification Rate of Agricultural Products

**Figure 3:**
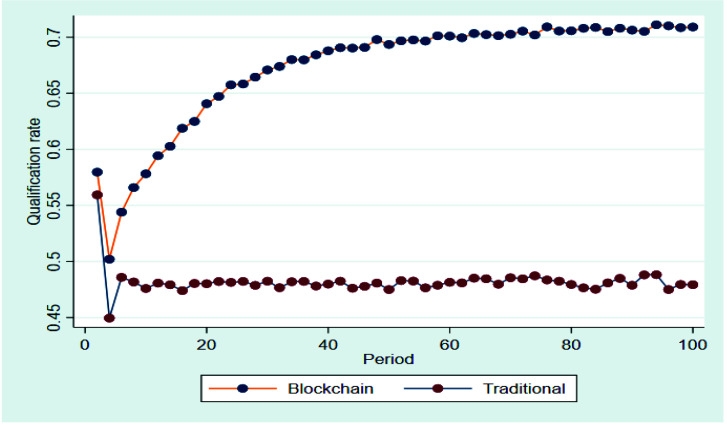
Qualification rate of agricultural products in the blockchain circulation chain and the traditional circulation chain.

Figure 3 shows that the qualification rate of agricultural products in the blockchain circulation chain increases quickly before the 50th period and increases slowly between the 51st and 75th periods. Despite of slight fluctuations, the qualification rate was maintained at high levels after the 75th period and reached approximately 71%. The qualification rate of agricultural products in the traditional circulation chain fluctuated considerably in the first two periods and was maintained at the relatively low levels of 47% to 48% after the second period, with occasional slight fluctuations. Therefore, the application of blockchain technology improved the qualification rate of agricultural products in the circulation system, which was clearly conducive to ensuring the quality and safety of agricultural products. H1 was tested.

### 5.2. Simulation of the Circulation Efficiency for Agricultural Products

**Figure 4:**
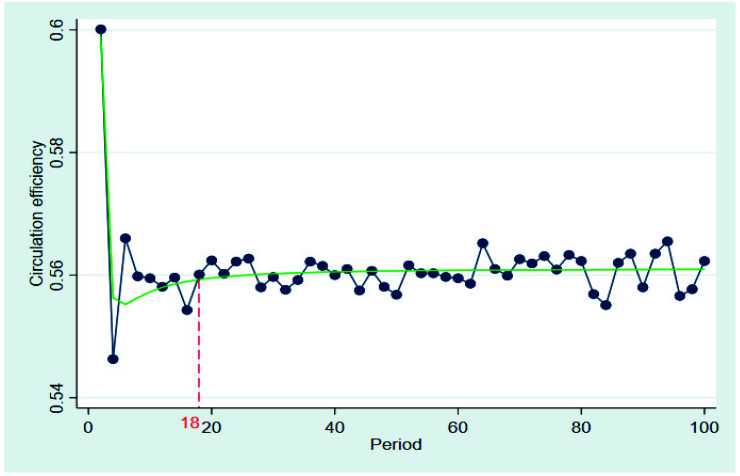
Circulation efficiency for agricultural products in traditional circulation chain.

Figure 4 shows the changes in the circulation efficiency of the traditional circulation chain over time, under the initial conditions with a certain supervision intensity. The circulation efficiency for agricultural products fluctuated considerably, especially in the first four periods, until the 18th period in the traditional distribution chain. After the 18th period, the circulation efficiency gradually maintained a relatively low level of approximately 0.56. The green line is a fitted curve of circulation efficiency over time in the traditional circulation chain; it can be seen that the circulation efficiency was basically stable at approximately 0.56.

**Figure 5:**
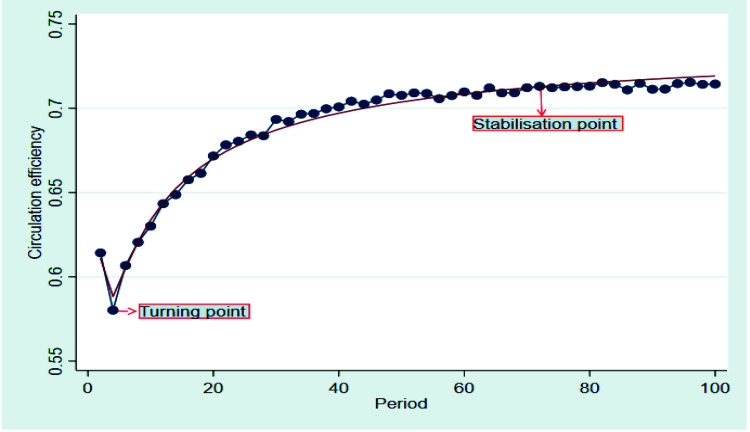
Circulation efficiency for agricultural products in blockchain circulation chain.

Figure 5 shows the changes in the circulation efficiency of the blockchain circulation chain over time, under the initial conditions with a certain supervision intensity. As shown in Figure 5, there was a turning point in the 4th period, when the circulation efficiency for agricultural products in the blockchain circulation chain changed from a rapid decrease to a slow increase. After the 4th period, the circulation efficiency was high and showed a rising trend over time, reaching the highest level in approximately the 70th period and then remaining stable; the circulation efficiency for the stable time in the blockchain circulation chain exceeded 0.7.

**Figure 6:**
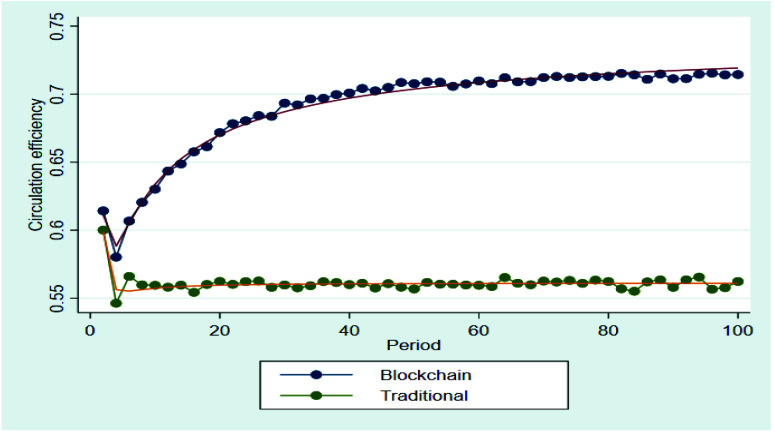
Circulation efficiency of the blockchain circulation chain and the traditional circulation chain.

Figure 6 compares the changes in circulation efficiency, over time, of the traditional circulation chain and the blockchain circulation chain after the introduction of blockchain technology. The results showed that the circulation efficiency in the traditional circulation chain remained at a low level after the 4th period, while the circulation efficiency in blockchain circulation chain grew consistently until the 70th period and then remained at a high level. Based on these results, we found that the introduction of blockchain technology had a positive impact on improving the circulation efficiency for agricultural products, because the introduction of blockchain technology changed the way of information was transmitted and improved the information asymmetry between buyers and sellers, which in turn altered the circulation mode of agricultural products and reduced the circulation links. H2 was tested.

### 5.3. Impact of Regulatory Intensity on the Circulation Efficiency and Qualification Rate for Agricultural Products

When other conditions remained unchanged, the impact of changes in regulatory intensity on the agricultural product circulation system was simulated by establishing different sampling probabilities. When the sampling probability was 0.1, the product qualification rate of the traditional circulation chain was stable at 9.06%, with a circulation efficiency of 0.1687. The qualification rate for products in the blockchain circulation chain was 47.09% and the circulation efficiency was 0.6942. When the sampling probability was increased to 0.3, the product qualification rate of the traditional circulation chain was stable at 38.76% and the circulation efficiency was 0.2501. For the blockchain circulation chain, the product qualification rate was 65.93% and the circulation efficiency was 0.7066 (Table 1). Overall, with a gradual increase in the probability of sampling inspection, the qualification rate of agricultural products increased rapidly in the traditional circulation chain and, accordingly, the circulation efficiency also increased. In contrast, the product qualification rate and the circulation efficiency in the blockchain circulation chain were always relatively high and only increased slightly with increased sampling probability. Therefore, the increase in regulatory intensity had an obvious effect on the traditional circulation chain, but a rather limited effect on the blockchain circulation chain, because the probability of random inspection directly affected the economic benefits of each agent and each subject changed behavioral decisions in the interests of economic benefits, thereby affecting the qualification rate and circulation efficiency for agricultural products. However, the blockchain technology itself had “internal regulation attributes” and, accordingly, external regulation had a relatively lower significant effect. We speculated that the introduction of blockchain technology can effectively reduce regulation costs and improve the government’s governance level for agricultural products.

## 6. Conclusions and Policy Implications

Increasing the qualification rate and circulation efficiency of agricultural products are key objectives in promoting quality products that are safe, people-oriented, and sustainable, with important significance for health. With the advent of the digital era, the successful integration of digital technology and agriculture has increased. With the advancement of digital technology, such as blockchain, the agriculture circulation industry is able to improve on the traditional model with a new model that is revolutionary. By combining the technical characteristics of blockchain and the operation mode of China’s agricultural product circulation system, this paper used agent-based modelling to establish a system that simulated the effect of blockchain technology on the agricultural product circulation system. This method provided answers to the following two questions: (1) Can the application of blockchain technology improve the qualified rate of agricultural products, thus ensuring the health of consumers? (2) Can the application of blockchain technology improve the circulation efficiency of agricultural products?

The simulation analysis revealed that blockchain technology can improve the qualification rate and ensure the quality and safety of agricultural products, as well as effectively enhance the circulation efficiency of the agricultural product circulation system and provide great benefits. The simulation experiments demonstrated that the increase in regulatory intensity has no significant impact on the blockchain circulation chain. Therefore, the introduction of blockchain technology can reduce regulation costs and improve governance to a certain extent.

The findings provide guidance on how to increase the qualification rate and circulation efficiency of agricultural products, and thus to ensure the health of the population. Therefore, four policy implications are proposed, based on this study. 

First, policy makers should pay increased attention to the application of blockchain technology in the circulation of agricultural products, increase investment in the development of high-tech in the circulation of agricultural products, and speed the formulation of standards for the application of blockchain technology in China. In addition, the government should improve the legal and technical rules of large-scale blockchain technology and regulate the consensus mechanism based on the applicable codes and algorithms. At the same time, we should strengthen the supervision of the agricultural information flow and establish an improved legal system to prevent information distortion. 

Second, in the context of building a large, unified national market, increased attention should be paid to the rural area, including the circulation system for agricultural products. We need to enhance the coordination of China’s various circulation entities, reduce circulation links, shorten circulation channels, and implement comprehensive circulation chain management. 

Third, the government should promote the extensive implementation of the optimization model, break the regional imbalance and uncoordinated status quo, and carry out high-quality management on the value-added process of agricultural products’ circulation in order to improve the overall efficiency of agricultural products’ circulation and promote the steady and rapid development of the rural economy. 

Finally, the application of blockchain technology in agriculture should be implemented gradually. The application of blockchain technology can be divided into an initial stage, a development stage, and a mature stage. In the initial stage, blockchain technology can mainly be applied to the circulation process of important agricultural products (e.g., rice and wheat). In the development stage, blockchain technology can be applied to the circulation process of strategic agricultural products, such as meat, cotton, and soybeans. In the mature stage, blockchain technology can be applied to the entire agricultural circulation system.

The research contributions of this paper include the following: (1) Theoretically, a logical framework for the influence of blockchain technology on the circulation system of agricultural products was constructed, and the influence of blockchain technology on the qualification rate and circulation efficiency of agricultural products was explored by a simulation analysis with agent-based modelling; these efforts expanded the relevant theories in the field of agricultural circulation. (2) Practically, this study helps in promoting the application of blockchain technology in the field of agriculture. With an increase in people’s expectations for the quality of agricultural products there will be more research in the future on blockchain technology in the field of agricultural products, including research on blockchain technology and agricultural machinery, the impact of blockchain technology on the production of agricultural products, and the applications of blockchain technology for the consumer.

There are some shortcomings in this article. The main shortcoming is that there are few applications of blockchain technology in the Chinese agricultural sector. In addition, the application of blockchain technology in the circulation of agricultural products is still immature and needs to meet certain requirements for basic hardware and software equipment. The decentralized nature of blockchain technology may also hinder operational efficiency. This article’s analysis of the behavior of the simulation agents was not sufficiently comprehensive. However, with the rapid development of blockchain technology, we believe that the circulation industry for agricultural products, based on blockchain technology, will continue to develop.

## Figures and Tables

**Table 1:** Impacts of changes in regulatory intensity on the product qualification rate and circulation efficiency.

Sampling Probability	Qualification Rate of Traditional Circulation Chain Products	Qualification Rate of Blockchain Circulation Chain Products	Circulation Efficiency of Traditional Circulation Chain	Circulation Efficiency of Blockchain Circulation Chain
0.1	0.0906	0.4709	0.1687	0.6942
0.2	0.2449	0.6102	0.2501	0.7009
0.3	0.3876	0.6593	0.3098	0.7066

## Data Availability

Data used in this research were provided by the Institute of Horticultural Economics, Huazhong Agricultural University. The datasets produced and analyzed for this study are available from the corresponding author(s) on reasonable request.
